# Minimally invasive resection of an extensive goiter with retrosternal extension: a case report and literature review

**DOI:** 10.1097/RC9.0000000000001052

**Published:** 2026-10-09

**Authors:** Alghaydaa Fouad Aldoughan, Abdulaziz Abdullah Alghamdi, Rakan Hassan Alzahrani, Abdullah Ahmed Alarfaj

**Affiliations:** a Otorhinolaryngology Department, King Abdulaziz Hospital, MNGHA, Al-Ahsa, Saudi Arabia; b Surgery Department, Almoosa Specialist Hospital, Al-Ahsa, Saudi Arabia; c Department of Surgery, King Abdulaziz Hospital, MNGHA, Al-Ahsa, Saudi Arabia; d Otorhinolaryngology Department, Ministry of Health, Aljaber ENT and Eye Hospital, Al-Ahsa, Saudi Arabia; e Otorhinolaryngology Unit, Surgery Department, Almoosa Specialist Hospital, Al-Ahsa, Saudi Arabia; f Otorhinolaryngology Unit, Department of Surgery, Medical College, King Faisal University, Al-Ahsa, Saudi Arabia

**Keywords:** case report, cervical incision, cervicomediastinal goiter, VATS

## Abstract

**Introduction and importance:**

Giant cervicomediastinal goiters compressing major vessels have traditionally required median sternotomy with a low cervical collar incision for complete resection. Although sternotomy provides optimal exposure, it is highly invasive and associated with increased postoperative morbidity. We report the successful resection of a giant cervicomediastinal goiter using a combined cervical and thoracoscopic approach, thereby offering a less invasive alternative while achieving favorable outcomes.

**Case presentation:**

A 41-year-old Saudi woman presented with anterior neck swelling due to a massive cervicomediastinal goiter compressing the left brachiocephalic vein near its junction with the superior vena cava (SVC). Contrast-enhanced CT demonstrated diffuse multinodular thyroid enlargement with retrosternal extension and significant tracheal and vascular compression. The goiter was successfully resected through a low cervical collar incision combined with a thoracoscopic approach, resulting in an uncomplicated recovery and early discharge.

**Clinical discussion:**

Median sternotomy provides excellent exposure for such masses but carries higher risks of infection, bleeding, and a prolonged hospital stay. Video-assisted thoracoscopic surgery (VATS) combined with thyroidectomy through a low cervical collar incision may allow a safer resection with fewer complications, as supported by previous case reports, comparative studies, and the present case.

**Conclusion:**

Combining a low cervical collar incision with a thoracoscopic technique is an efficient, minimally invasive method for resecting large goiters, ensuring optimal access and improved surgical outcomes.

## Introduction

Benign multinodular goiter is the most prevalent endocrine disorder, particularly in iodine-deficient regions, affecting up to 5% of populations in nonendemic areas and 15% in endemic areas^[^1^]^. Surgery is indicated for compressive symptoms, suspected malignancy, substantial substernal extension, refractory hyperthyroidism, or cosmetic concerns^[^2,3^]^. Retrosternal goiter, defined as thyroid extension below the thoracic inlet or with more than 50% of its volume below this level, can compress the trachea, esophagus, and great vessels^[^4^]^.

HIGHLIGHTS

- A combined cervical and thoracoscopic approach was successfully used to remove a giant cervicomediastinal goiter.
- This approach may reduce postoperative pain and provide better cosmetic outcomes.
- Thoracoscopic visualization provided controlled dissection and hemostasis during the procedure.
- This case emphasizes the importance of careful preoperative imaging, airway planning, and multidisciplinary collaboration.
- A minimally invasive cervico-thoracoscopic approach can be considered for selected retrosternal goiters when imaging shows favorable dissection planes and no frank invasion.

Most substernal goiters remain connected to the cervical gland and can be resected via a low cervical collar incision, though 4–12% require additional thoracic access. Video-assisted thoracoscopic surgery (VATS) combined with a cervical incision offers a less invasive alternative with better exposure, reduced morbidity, and improved cosmesis^[^5^]^, and has shown promising outcomes even in high-risk patients^[^6^]^. We present a 41-year-old woman with a giant cervicomediastinal goiter resected using this combined approach. This report follows the SCARE checklist^[^7^]^.

## Case report

A 41-year-old previously healthy woman developed progressive dysphagia over 4 months, followed by mild dyspnea and anterior neck swelling for 1 month, and presented to the ENT clinic on 29 June 2024. The earlier onset of compressive symptoms relative to visible swelling suggested gradual enlargement of a predominantly deep cervical or retrosternal component rather than true rapid growth. She denied weight loss or other swellings. Examination revealed a soft, non-tender thyroid enlargement approximately 4 cm in its greatest dimension, without skin changes. Thyroid function tests (TSH, free T4) were normal.

Neck ultrasound showed a diffusely enlarged, multinodular gland involving both lobes and the isthmus, with retrosternal extension. The dominant right-lobe nodule measured 24 × 24 mm (TI-RADS 3); a similar isthmic nodule measured 26 × 22 mm. No cervical lymphadenopathy was seen. Fine-needle aspiration of the right-lobe and isthmic nodules revealed benign follicular nodules with cystic change (Bethesda II); the retrosternal component was not sampled owing to its depth.

Contrast-enhanced CT confirmed a diffusely enlarged gland with multiple nodules. The largest lesion arose from the right lower pole and extended into the isthmus (5.2 × 5.5 × 7.2 cm), with retrosternal extension reaching approximately 2.2 cm below the manubrium and severe tracheal compression(narrowest anteroposterior diameter approximately 5 mm)without complete obstruction (Fig. 1). A right upper paratracheal mediastinal lesion (5.8 × 4.2 × 4.9 cm) was located just inferior to the thyroid nodule, and its proximity and enhancement were most consistent with extension of a mediastinal goiter. It partially encased the superior vena cava (SVC) over approximately 180 degrees below the innominate artery, an important landmark for cervical feasibility, with mild luminal narrowing but no vascular invasion or thrombosis. The extension crossed the midline, significantly compressing the left brachiocephalic vein, though no clinical signs of venous obstruction were present. MRI confirmed these findings, demonstrating a dominant isthmic nodule (7.8 × 5.8 cm), mass effect without vascular invasion, and a patent airway (Fig. 2).

**Figure 1.**
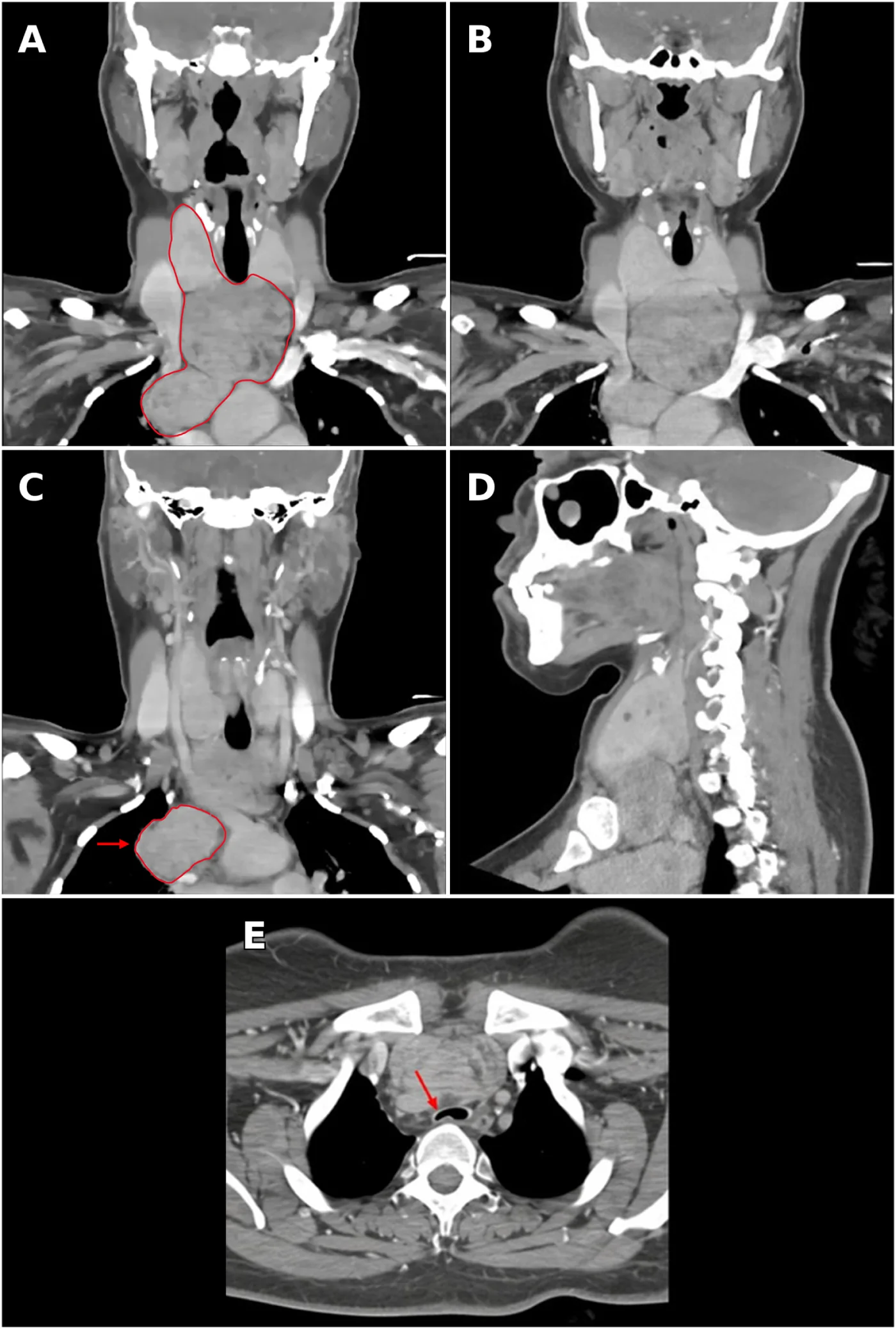
Contrast-enhanced CT images of the neck demonstrating a diffusely enlarged thyroid gland with multiple nodules of varying sizes. The largest lesion arises from the lower pole of the right thyroid lobe and extends into the isthmus. (A) Coronal CT reconstruction showing a diffusely enlarged thyroid gland with largest lesion outlining the multinodular goiter (red outline). (B) Coronal non-outlined view illustrating the extent of the cervicomediastinal mass and its relation to adjacent structures. (C) A heterogeneously enhancing soft-tissue mass in the right upper paratracheal region causes mild narrowing of the superior vena cava (SVC) lumen, along with significant compression of the left brachiocephalic vein (red arrow). (D) Notable tracheal compression is seen, with the narrowest anteroposterior diameter measuring approximately 5 mm. (E) Axial CT at the thoracic inlet, demonstrating the narrowest tracheal caliber, with an AP diameter of approximately 5 mm(red arrow).

**Figure 2.**
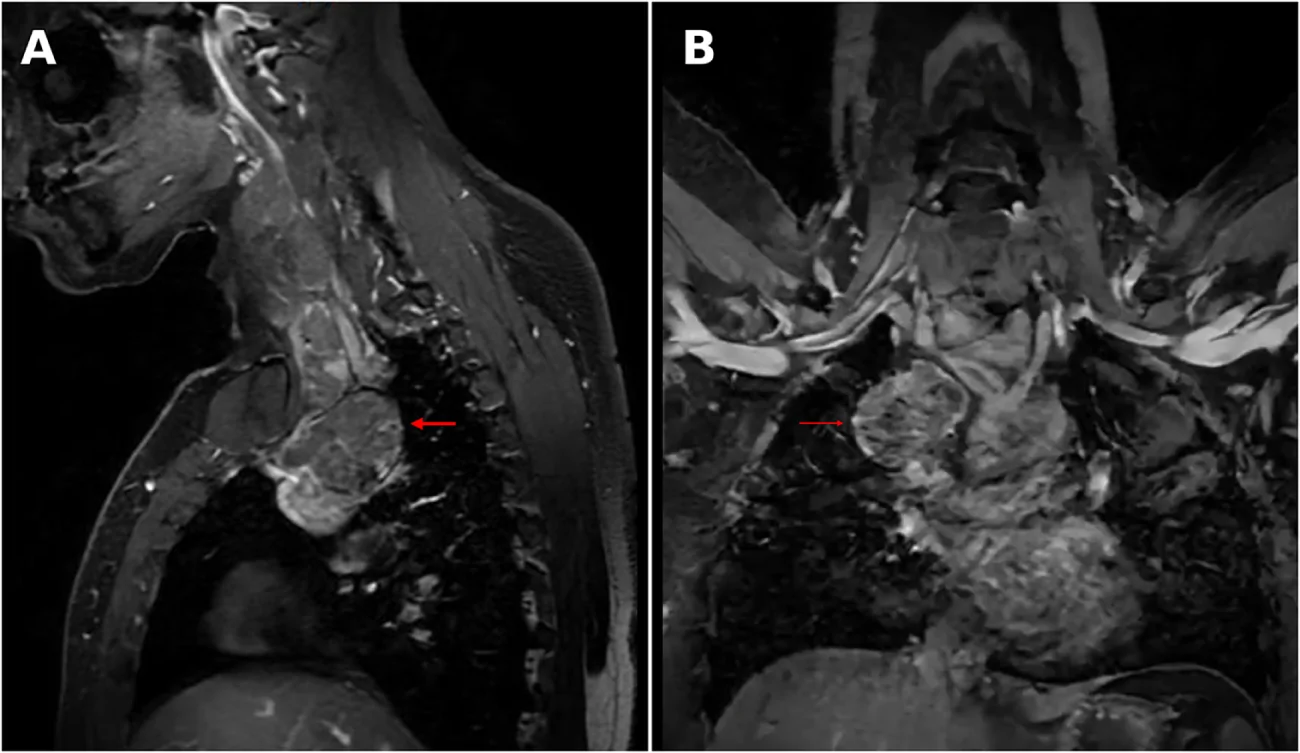
Sagittal and coronal MRI images demonstrate mediastinal extension with mass effect on adjacent vascular structures, without evidence of vascular occlusion.

After multidisciplinary evaluation, a low cervical collar incision combined with thoracoscopic resection was planned. Preoperative laryngoscopy showed no significant upper airway obstruction. Given the tracheal compression, an awake fiber-optic intubation with a single-lumen tube was performed to maintain spontaneous ventilation.

For the right hemithyroidectomy and isthmusectomy, a 7-cm low cervical collar incision was made, and a subplatysmal flap was raised from the hyoid bone to the sternal notch. The left lobe was preserved; the right lobe, isthmus, and mediastinal component were resected, with the recurrent laryngeal nerve identified and preserved using intraoperative nerve stimulation. Intraoperative ultrasound confirmed residual mediastinal extension that could not be safely delivered via a cervical approach. A neck drain was placed, and the incision was closed.

The single-lumen tube was exchanged for a double-lumen tube for one-lung ventilation, and the patient was repositioned in left lateral decubitus. Thoracoscopic access used three 1-cm ports (camera at the mid-axillary line, 7th intercostal space; working and assistant ports at the 5th intercostal space). The LigaSure Maryland device (Covidien, Mansfield, MA, USA) was used to open the mediastinal pleura and dissect the mediastinal component from the trachea and SVC near the brachiocephalic trunk. Adjacent tissues were markedly thinned and vascular, raising bleeding risk, but hemostasis was achieved with blunt dissection and cautery. A 28-Fr chest tube was placed, and the wound was closed.

The patient was extubated after laryngeal assessment of vocal cord function. Total operative time was 4 hours and 50 minutes (2 hours 30 minutes cervical, 50 minutes repositioning and tube exchange, 1 hour 30 minutes thoracoscopic), with minimal blood loss. She was monitored for 2 nights, had drains removed sequentially, and was discharged on postoperative day 2. Histopathology confirmed a giant nodular goiter measuring approximately 10 × 5 cm (Fig. 3). At 1-year follow-up, she remained clinically stable with preserved voice, normal serum calcium, normal thyroid function without replacement therapy, and no recurrence.

**Figure 3.**
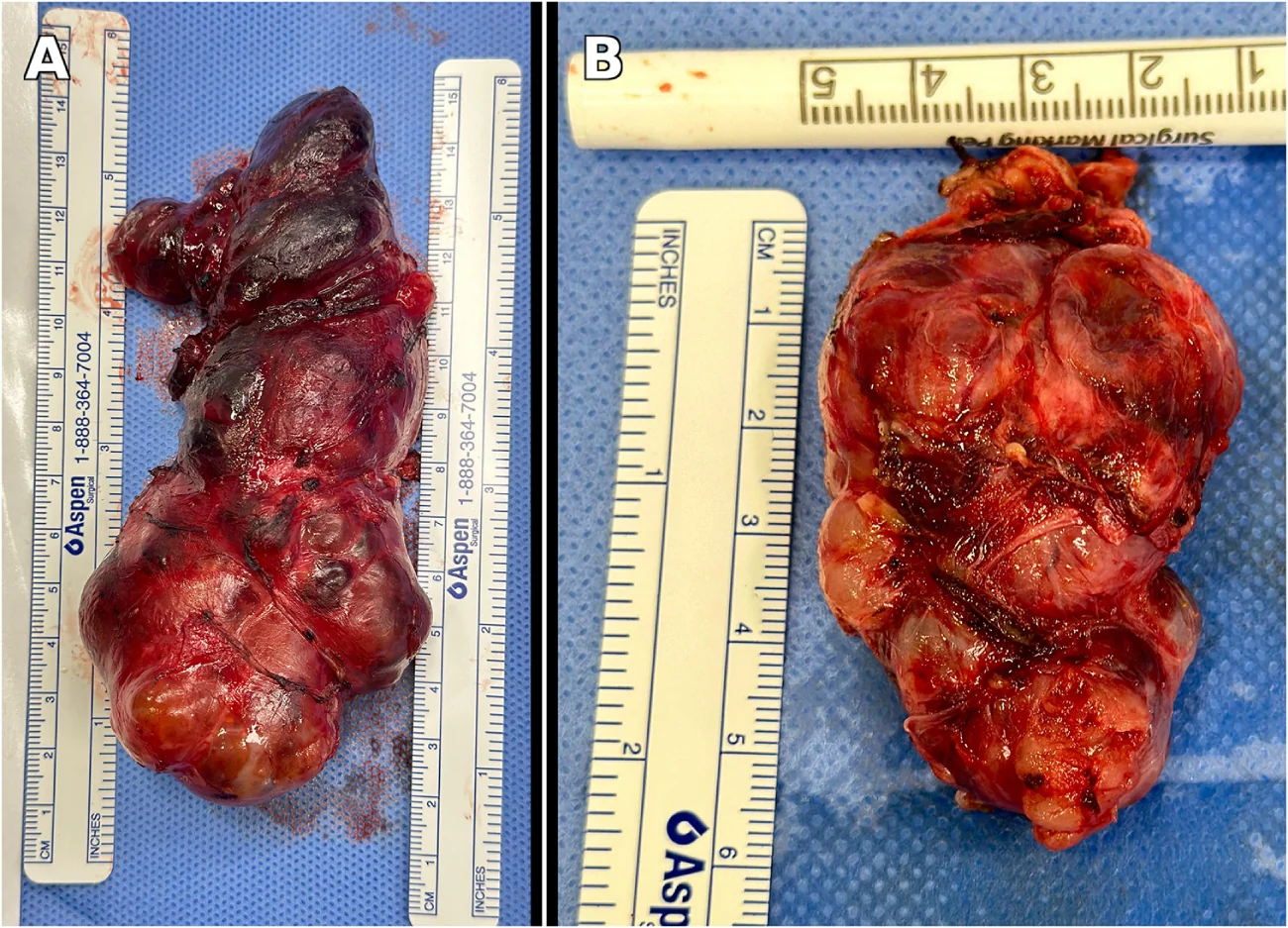
(A and B) Gross specimen of the resected goiter measuring approximately 10 × 5 cm(rulers in cm).

## Discussion

Retrosternal goiters are generally defined as thyroid enlargement that extends beyond the thoracic inlet into the mediastinum and can cause compressive symptoms, such as dysphagia and dyspnea. Surgical resection is recommended, typically for symptomatic patients or those with suspected malignancy. Most (approximately 85–95%) are excised via a cervical incision alone, while a minority require median sternotomy^[^8^]^.

Although sternotomy provides excellent exposure for intrathoracic goiters extending beyond the aortic arch or enveloping great vessels, it carries greater surgical trauma and complications, including prolonged recovery, postoperative pain, wound dehiscence, sternal infection, and significant bleeding^[^9^]^. This has driven demand for less invasive approaches that achieve complete resection without opening the sternum.

VATS combined with a low cervical collar incision accesses mediastinal thyroid tissue through small ports, providing adequate visualization while avoiding thoracotomy or sternotomy, and has emerged as an effective alternative for selected large retrosternal goiters. Morimoto *et al* (2023) removed a giant retrosternal goiter extending to the tracheal bifurcation with a short hospital stay and rapid recovery^[^10^]^, and Shigemura *et al* resected a huge intrathoracic goiter in a high-risk patient using VATS with a small supraclavicular window and minimal complications^[^6^]^. Comparative data support these advantages: Rebei *et al* (2022) found thoracoscopic resection of anterior mediastinal tumors produced fewer complications and a shorter stay than sternotomy (mean 4 vs 6 days), with the sternotomy group showing higher rates of bleeding, infection, and three deaths^[^9^]^. Although the data are not thyroid-specific, this reinforces the broader benefits of minimally invasive mediastinal access^[^9,10^]^. Traditional open approaches remain appropriate for dense adherence, frank vascular invasion, posterior mediastinal extension, or high suspicion of malignancy^[^11^]^.

Each retrosternal goiter requires multidisciplinary evaluation. Anterior goiters above the aortic arch can often be delivered cervically, whereas those extending beyond the arch, into the posterior mediastinum, or closely related to major vessels more often require an extracervical approach^[^12^]^. In our case, the mediastinal component reached the fourth thoracic vertebra and lay adjacent to the SVC and left brachiocephalic vein, features that traditionally raise concern for sternotomy. However, CT and MRI showed mass effect only, without vascular invasion, thrombosis, or malignant features, suggesting a safe dissection plane. Thoracoscopy allowed controlled dissection from the trachea, SVC, and vessels, with LigaSure-assisted hemostasis. This case supports the view that a combined cervical–VATS approach may be appropriate for selected cervicomediastinal goiters when imaging demonstrates favorable planes and no frank invasion, even when some traditional sternotomy criteria are present.

## Conclusion

A low cervical collar incision combined with VATS offers a suitable alternative to sternotomy for selected patients with favorable anatomical and pathological features. It may allow complete resection while reducing postoperative morbidity, hospital stay, and cosmetic burden and should be considered during multidisciplinary planning when anatomy permits controlled dissection.

## Data Availability

The data that support the findings of the this study are available from the corresponding author upon reasonable request.

## References

[1] Lang BH-H Lo C-Y. Total thyroidectomy for multinodular goiter in the elderly. Am J Surg 2005;190:418–23. 10.1016/j.amjsurg.2005.03.029 16105529

[2] Mauriello C Marte G Canfora A. Bilateral benign multinodular goiter: what is the adequate surgical therapy? A review of literature. Int J Surg 2016;28:S7–S12. 10.1016/j.ijsu.2015.12.041 26708850

[3] Barczyński M Konturek A Stopa M. Total thyroidectomy for benign thyroid disease: is it really worthwhile? Ann Surg 2011;254:724–29. 10.1097/SLA.0b013e3182360118 22005150

[4] Abdullah AS Bahjat AS Mohammed AA. Huge toxic goiter extending to the posterior mediastinum: case report with literature review. Int J Surg Case Rep 2019;62:69–72. 10.1016/j.ijscr.2019.08.016 PMC6717091 31454617

[5] Gupta P Lau KKW Rizvi I. Video assisted thoracoscopic thyroidectomy for retrosternal goitre. Ann R Coll Surg Engl 2014;96:606–08. 10.1308/003588414X14055925058634 PMC4474103 25350184

[6] Shigemura N Akashi A Nakagiri T. VATS with a supraclavicular window for huge substernal goiter: an alternative technique for preventing recurrent laryngeal nerve injury. Thorac Cardiovasc Surg 2005;53:231–33. 10.1055/s-2005-837677 16037869

[7] SCARE Group. Kerwan A Al-jabir A Mathew G. Revised Surgical Case Report (SCARE) guideline: an update for the age of Artificial Intelligence. Prem J Sci 2025;10:100079.

[8] Nesti C Wohlfarth B Borbély Y. Modified thoracoscopic-assisted cervical resection for retrosternal goiter: case report. Front Surg 2021;8:695963. 10.3389/fsurg.2021.695963 PMC8219883 34179070

[9] Rebei M Abdennadher M Abdelkebira A. VATS or sternotomy in mediastinal tumors? Disorders of the Mediastinum Chest 2022;161:A266.

[10] Morimoto J Yamanaka T Yusa J. Successful excision of a giant cervical mediastinal goiter by cervical collar incision and a thoracoscopic approach: a case report. Gen Thorac Cardiovasc Surg Cases 2023;2:41. 10.1186/s44215-023-00058-x PMC11533441 39516959

[11] Magistrelli P. Treatment of cervico-mediastinal goiters. World J Surg Proced 2013;3:37–40.

[12] Mercante G Gabrielli E Pedroni C. CT cross-sectional imaging classification system for substernal goiter based on risk factors for an extracervical surgical approach. Head Neck 2011;33:792–99. 10.1002/hed.21539 20737483

